# Cannabinoid system and cyclooxygenases inhibitors

**Published:** 2011-02-25

**Authors:** H Păunescu, OA Coman, L Coman, I Ghiţă, SR Georgescu, F Drăia, I Fulga

**Affiliations:** * Department of Pharmacology and Pharmacotherapy, ‘Carol Davila’ University of Medicine and PharmacyRomania; ** Department of Physiology, ‘Carol Davila’ University of Medicine and PharmacyRomania; *** Department of Dermatology, ‘Carol Davila’ University of Medicine and PharmacyRomania; **** Department of Anatomy, ‘Carol Davila’ University of Medicine and PharmacyRomania

**Keywords:** cannabinoids, NSAIDs, cyclooxygenase, analgesia

## Abstract

**Rationale.** The cannabinoid system consists of a complex array of receptors, substances with agonist/antagonist properties for those receptors, biosynthetic machineries and mechanisms for cellular uptake and degradation for endocannabinoids. This system is in interrelation with other systems that comprise lipid mediators like prostaglandins/leukotrienes systems. A clear antagonist, additive or synergic effect of nonsteroidal anti–inflammatory drugs (NSAIDs)–cannabinoid associations was not yet demonstrated. **Aim.** The present study tried to summarize the existent data on NSAIDS-cannabinoid system interactions.

**Methods and results** A bibliographic research in Medline, Scirus, Embase was made using as keywords cannabinoid, nonsteroidal anti–inflammatory drugs, aspirin, ibuprofen, flurbiprofen, diclofenac, indomethacin, acetaminophen, coxibs, antinociceptive, antinociception, analgesia

**Discussions**A systematization of the results focusing on the NSAIDs drugs interaction with the cannabinoid system was presented. Out of all the substances analyzed in the present review, acetaminophen was studied the most regarding its interferences with the cannabinoid system, mainly due to contradictory results.

**Conclusions** Some NSAIDs have additional influences on the cannabinoid system either by inhibiting fatty acid amide hydrolase (FAAH) or by inhibiting a possible intracellular transporter of endocannabinoids. All the NSAIDs that inhibit COX2 can influence the cannabinoid system because a possible important degradative pathway for anandamide and 2–arachidonoyl glycerol might involve COX 2. One of the causes for the variety of experimental results presented might be due to pharmacokinetic mechanisms, depending on the route of administration and the dose

**Abbreviations**delta9 THC, (–)–(6aR,10aR)–6,6,9–trimethyl–3–pentyl–6a,7,8,10a–tetrahydro–6H–benzo[c]chromen–1–ol; delta9–THC–11–oic acid, 1–hydroxy–6,6–dimethyl–3–pentyl–6a,7,8,10a–tetrahydrobenzo[c] chromene–9–carboxylic acid; Anandamide, (5Z,8Z,11Z,14Z)–N–(2–hydroxyethyl)icosa–5,8,11,14–tetraenamide; Methanandamide, (5Z,8Z,11Z,14Z)–N–[(2R)–1–hydroxypropan–2–yl]–icosa–5,8,11,14–tetraenamide; 2–AG, 1,3–Dihydroxy–2–propanyl (5Z,8Z,11Z,14Z)–5,8,11,14–eicosatetraenoate; HU 210, (6aR,10aR)– 9–(Hydroxymethyl)–6,6–dimethyl–3–(2–methyloctan–2–yl)–6a,7,10,10a–tetrahydrobenzo [c]chromen–1–ol; SR141716A, 5–(4–Chlorophenyl)–1–(2,4–dichloro–phenyl)–4–methyl–N–(piperidin–1–yl)–1H–pyrazole–3–carboxamide; SR144528, N–[(1S)–endo–1,3,3–trimethyl bicyclo [2.2.1] heptan–2–yl]–5–(4–chloro–3–methylphenyl)–1–(4–methylbenzyl)–pyrazole–3–carboxamide; AM251, 1–(2,4–dichlorophenyl)–5–(4–iodophenyl)–4–methyl–N–(1–piperidyl)pyrazole–3–carboxamide; AM 404, (5Z,8Z,11Z,14Z)–N–(4–hydroxyphenyl)icosa–5,8,11,14–tetraenamide; WIN 55,212–2, (R)–(+)–[2,3–Dihydro–5–methyl– 3–(4–morpholinylmethyl)pyrrolo [1,2,3–de]–1,4–benzoxazin–6–yl]–1–napthalenylmethanone; AM 281, N–(morpholin–4–yl)–5–(4–iodophenyl)–1–(2,4–dichlorphenyl)–4–methyl–1H–pyrazole–3–carboxamide; AM 630, [6–Iodo–2–methyl–1–[2–(4–morpholinyl)ethyl]–1H–indol–3–yl](4–methoxyphenyl)methanone; Ibu Am–5, N–(3–methylpyridin–2–yl)–2–(4'–isobutylphenyl)propionamide; CP 55, 940, 2–[(1R,2R,5R)–5–hydroxy–2–(3–hydroxypropyl) cyclohexyl]–5–(2–methyloctan–2–yl)phenol; NS–398, N–[2–(Cyclohexyloxy)–4–nitrophenyl]methanesulfonamide; SC–560, 5–(4–chlorophenyl)–1–(4–methoxyphenyl)–3–trifluoromethylpyrazole; AM 1241, (3–iodo–5–nitrophenyl)–[1–[(1–methylpiperidin–2–yl)methyl]indol–3–yl]methanone; Met F AEA, 2–methyl–arachidonyl–2'–fluoro–ethylamide; PMSF, Phenylmethylsulfonyl fluoride; URB 597, [3–(3–carbamoylphenyl)phenyl] N–cyclohexylcarbamate; TRPV1, Transient receptor potential vanilloid type 1; CGRP, Calcitonin gene related peptide; COX1, cyclooxygenase type 1; COX2, cyclooxygenase type 2; CB1R, cannabinoid receptor type 1; CB2R, cannabinoid receptor type 2; FAAH, fatty acid amide hydrolase; NSAIDs, nonsteroidal anti–inflammatory drugs; p.o., per os; i.p., intraperitoneally; i.th., intrathecally; s.c. subcutaneously; i.pl., intraplantar; i.v., intravenously; CB cannabinoid.

## Introduction

Only in 1964 when Ganoi and Mechoulam identified delta9 tetrahydrocannabinol (delta9 THC) being the main psychotropic agent from *Cannabis sativa* the researches in the ‘field’ of cannabinoids gain scale. Many efforts to discover the substrate of psychotropic and analgesic effects of delta9 THC were made. The discovery of cannabinoid receptors and endogenous cannabinoids (endocannabinoids) came about twenty years later. The two main endocannabinoids discovered were, in order, anandamide (arachidonoyl ethanolamine) and 2–arachidonoyl glycerol.

Cannabinoid system consists of a complex array of receptors, substances with agonist/antagonist properties for those receptors, biosynthetic machineries and mechanisms for cellular uptake and degradation for endocannabinoids. It might represent a new target for drugs that produce analgesia, attenuation of nausea and vomiting in cancer chemotherapy, reduction of intraocular pressure, appetite stimulation in wasting syndromes, relief from muscle spasms/spasticity in multiple sclerosis and decreased intestinal motility.

The positive effects are often accompanied by adverse reactions like alterations in cognition and memory, dysphoria/euphoria, and sedation [1].

The endocannabinoid system is in interrelation with other systems that comprise lipid mediators like prostaglandins/leukotrienes systems [2]. Nowadays it is well known that cyclooxygenase type 2 (COX2) actions both on arachidonic acid, resulting prostaglandins and other eicosanoids, and on endocannabinoids (anandamide and 2–arachidonoyl glycerol), resulting prostamides and prostaglandin glycerol esters. It is not surprising that these substances have different pharmacological properties than the amides or the esters from which they are derived. From this point of view the inhibition of cyclooxygenases, especially COX2, might have many influences at the level of central nervous system or in immune cells (two of the main domains that are rich in cannabinoid receptors and in cannabinoids). The cyclooxygenase products of endocannabinoids were reviewed elsewhere [3–7] and will not make a subject for this paper.

The cannabinoid receptors and endocannabinoids. The human cannabinoid receptor 1 (CB1R) was cloned by Gerrard et al. (1991). CB1 receptors are coupled with Gi/Go proteins and are serpentine receptors. Through G protein action the activity of adenylyl cyclase is diminished, which leads to a decrease of cAMP level. The activity of some ionic channels is also modulated.

The human cannabinoid receptor 2 (CB2R) was first identified in man in 1993. CB2 receptors are coupled with Gi/Go type proteins. Unlike CB1 receptors, the CB2 ones do not seem to be coupled to ionic channels. They are coupled with intracellular signalization pathways associated to MAP kinase.

Another two serpentine receptors, classified among orphan receptors because, when discovered, there did not exist a specific ligand to bind them, are supposed to be cannabinoid receptors. These two receptors are still named GPR55 and GPR119. Another receptor for anandamide is the transient receptor potential vanilloid1 receptor (TRPV1), the receptor for capsaicin [1].

Anandamide and especially 2–arachidonoyl glycerol can function as retrograde synaptic messengers. They are released from postsynaptic neurons and travel backward across synapses, activating CB1 on presynaptic axons and suppressing neurotransmitter release. Cannabinoids may affect memory, cognition, and pain perception by means of this cellular mechanism [8].

Endogenous ligands for CB receptors discovered until now are eicosanoids: N–arachidonoylethanolamide (anandamide), 2–arachidonoyl glycerol, noladin ether, O–arachidonoylethanolamine (virodhamide) and N–arachidonoyldopamine.

Anandamide, 2–arachidonoyl glycerol, and N–arachidonoyldopamine are susceptible to degradation by fatty acid amide hydrolase (FAAH), although a second enzyme, monoacylglycerol lipase, catalyzes hydrolysis of 2–arachidonoylglycerol in vivo [1].

Numerous substances with cannabinoid properties were described. They might act as full or partial agonists, antagonists or inverse agonists, neutral antagonists [9], or may increase the endocannabinoids level (FAAH inhibitors, cellular uptake of cannabinoids inhibitors). Some of them are presented in Table 1 [1]. 

**Table 1:** Classification of substances that influence endocannabinoid system

	Cannabinoid receptor agonists	
Classical cannabinoids	delta9 THC	partial agonist of CB1R and CB2R
	HU 210	complete agonist of both CB1R and CB2R
Non–classical cannabinoids	CP–55, 940	complete agonist of both CB1R and CB2R
Specific CB–2 receptor agonist	AM 1241	
Aminoalkylindoles	WIN–55, 212–2	complete agonist of both CB1R and CB2R, slightly selective for CB2R
Eicosanoids	Anandamide (AEA) R–(+)–methanandamide	partial agonist of both CB1R and CB2R and TRPV1 agonist
	Met F AEA 2–AG	full agonist of both CB1R and CB2R
	Cannabinoid receptor antagonists/inverse agonists	
Diarylpyrazoles and other derivatives	SR141716A [rimonabant], AM 251, AM281	selective CB1R blockers
	SR144528, AM 630	selective CB2R blockers
	Uptake blockers: AM 404	
	FAAH inhibitors: PMSF, URB 597	

Cyclooxygenases inhibitors or nonsteroidal anti–inflammatory drugs (NSAIDs) are a heterogeneous group of substances that block either the cyclooxygenase site of enzyme cyclooxygenase type 1 or 2 (COX 1 and COX 2, respectively), or its peroxidase site [10,11]. In the first category can be mentioned ibuprofen, diclofenac, indomethacin, coxibs (rofecoxib, celecoxib) and in the second category might be included acetaminophen and metamizole sodium. 

## Meth

A systematic analysis of data from existing literature databases Medline, Pubmed, Embase, Scirus up to 31.08.2010 was performed. Initial selection of articles was made using as key words cannabinoid AND (nonsteroidal anti–inflammatory drugs OR aspirin OR ibuprofen OR flurbiprofen OR diclofenac OR indomethacin OR acetaminophen OR coxibs) taking into account articles in abstract and full text from clinical and preclinical studies. 225 articles, published after the year 1972 to date, out of which 199 in full text and 26 in abstract, were found. The search area was reduced by introducing new keywords: antinociceptive OR antinociception OR analgesia. References to all relevant articles were examined to include all relevant reports and review sites on the subject. The study included data in English and French. Following the final selection, 24 items were retained in the study considering the exclusion criteria (analytical interference in the determination of cannabinoids and NSAIDs). The 24 studies that emphasized the interactions between the endocannabinoid system or exogenous cannabinoids and NSAIDs, especially on the analgesic effect, were analyzed in terms of types of cannabinoid receptors or of the endocannabinoids involved. Another aim was to elucidate the mechanism of action of cyclooxygenase inhibitors and their interactions with exogenous cannabinoid agonists.

## Results

A systematization of the data found in the articles studied are presented in  Table 2.

**Table 2:** Synopsis of data collected from 25 studies on NSAIDs and cannabinoid system interactions

No.	Cyclooxygenase pathway (prostaglandins precursors, prostaglandins, COX inhibitors)	Cannabinoid (agonists, antagonists) administered	Experimental method used	The results of the study	Discussion	Authors
1	Indomethacin (p.o.)	delta9–THC delta9–THC–11–oic acid (p.o.)	The hot plate test (in mice)	10 min after delta9–THC administration, a pronounced hyperalgesia was seen. Hyperalgesia could be inhibited by prior administration of either indomethacin or delta9–THC–11–oic acid.	The metabolite delta9–THC–11–oic acid inhibited eicosanoid synthesis whereas the parent drug (delta9–THC) elevated tissue levels of prostaglandins	Burstein SH, et al FASEB J. 1988 Nov;2(14):3022–6. [12]
2	Ibuprofen, aspirin, sulindac, acetaminophen, ketoprofen, naproxen		Rat cerebellar membrane preparation	The potency of ibuprofen as an inhibitor of anandamide metabolism was of the same magnitude as required for inhibition of COX2. Aspirin, sulindac, acetaminophen, ketoprofen and naproxen did not inhibit the anandamide metabolism.	The metabolism of anandamide might be affected, following the therapeutic doses of ibuprofen.	Fowler CJ, et al. Pharmacol Toxicol. 1997 Feb;80(2):103–7 [13]
3	Indomethacin (i. th.)	HU–210 (p.o. and i.th.)	Tail flick and formalin test (in mice) Spinal microdialysis	Indomethacin reduced the HU 210 effect on pronociceptive prostaglandins production but did not potentiate the analgesic effect of HU–210	HU–210 showed analgesic properties that are independent of its influence on the prostaglandin pathway.	Guhring H, et al. Eur J Pharmacol. 2001 Oct 19;429(1–3):127–34 [14]
4	Aspirin, indomethacin, celecoxib, ketorolac, acetaminophen diclofenac (p.o.)	delta9–THC, anandamide, arachidonic acid, ethanolamine, methanandamide, SR141716A,SR144528(i.p.)	The phenylbenzo–quinone writhing test (in mice)	After chronic treatment with delta9–THC the analgesic effect of diclofenac and acetaminophen decreased while the effect of aspirin, indomethacin, celecoxib, ketorolac was not detected. Chronic treatment with methanandamide did not alter the analgesic effects of the NSAIDs tested. Neither SR141716A, SR144528 blocked the effects of the NSAIDs tested.	The alteration of NSAIDs effects was not due to chronic administration of delta9–THC and might be due to pharmacokinetic mechanisms (some metabolites of delta9–THC might interfere with NSAIDs). Also it was stated that NSAIDs are not acting directly or indirectly at either the CB1R or CB2R.	Anikwue R, et al J Pharmacol Exp Ther. 2002 Oct;303(1):340–6 [15]
5	Indomethacin Prostaglandin E2(i. th)	AM251 (i.th.)	The formalin test (in spinally micro–dialyzed mice)	Indomethacin–induced analgesia was reversed by co–administration of AM251, but not by co–infusion of prostaglandin E2.	Indomethacin might acted by stimulation of CB1R or by increasing the level of endocannabinoids	Guhring H, et al. Eur J Pharmacol. 2002 Nov 15;454(2–3):153–63 [16]
6	Prostaglandin E2 Flurbiprofen (i. th)	AM251 (i. th)	The formalin test (in rats)	The analgesic effect of flurbiprofen (i.th.) was reversed by the co–administration of AM–251, but not by prostaglandin E2	Endocannabinoids played a major role in mediating flurbiprofen–induced analgesia	Ates M, et al. Eur J Neurosci. 2003 Feb;17(3):597–604 [17]
7	Flurbiprofen Prostaglandin E2 (i.th.)	delta9– THC AM251 (i.th.)	The spinal super–perfusion model (in rats)	delta9 THC inhibited capsaicin induced CGRP release. Similarly, flurbiprofen inhibited spinal CGRP release. This inhibition was reversed by AM–251, but not by co–administration of prostaglandin E2	The mechanism for flurbiprofen inhibitory effect on spinal CGRP release might be the shift of arachidonic acid metabolism towards endocannabinoids formation	Seidel K, et al. Neurosci Lett. 2003 Feb 27;338(2):99–102 [18]
8	Acetaminophen		Tissue Homogenate Experiments (Rat Purified FAAH Enzyme Assay, etc)	Acetaminophen, following deacetylation to p– aminophenol, was conjugated with arachidonic acid (FAAH dependent) to form the potent TRPV1 agonist AM404 that inhibited purified COX–1 and COX–2 and synthesis of prostaglandins	The study provided a molecular mechanism for the occurrence of the analgesic metabolite AM404 in the nervous system following treatment with acetaminophen	Hogestatt ED, et al. J Biol Chem. 2005 Sep 9;280(36):31405–12 [19]
9	Ketorolac (s.c)	WIN55,212–2 (s.c.)	The acetic acid–induced–writhing test (in mice)	WIN 55,212–2 and ketorolac either alone or in combination produced dose dependent analgesia in the writhing test.	Isobolographic analysis showed additive interactions between WIN 55,212–2 and ketorolac.	Ulugol A, et al. Anesth Analg. 2006 Feb;102(2):443–7 [20]
10	Acetaminophen (p.o)	AM281 SR141716A (i.p.)	The hot plate test in rats	The analgesic activity of acetaminophen is antagonized by AM281 and SR 141716A	Paracetamol–induced analgesia might involve the cannabinoid system.	Ottani A, et al. Eur J Pharmacol. 2006 Feb 15;531(1–3):280–1 [21]
11	Ibuprofen (i.pl.)	Anandamide (i.pl.) AM251 AM630	the formalin test (in rats)	Analgesic interaction between anandamide and ibuprofen was synergistic and completely antagonized by AM251 but only partially inhibited by AM630.	The combination of anandamide with ibuprofen produced synergistic analgesic effects involving both cannabinoid CB1R and CB2R.	Guindon J, et al. Pain. 2006 Mar;121(1–2):85–93 [22]
12	Ibuprofen Rofecoxib (in the hind paw)	Anandamide AM251 AM630 (in the hind paw)	Evaluation of mechanical allodynia and thermal hyperalgesia in neuropathic rats	Anandamide, ibuprofen, rofecoxib and their combinations significantly decreased mechanical allodynia and thermal hyperalgesia. The effects of these NSAIDs were not antagonized by AM251 or AM630.	Locally injected anadamide, ibuprofen, rofecoxib and their combinations decreased pain behavior in neuropathic animals.	Guindon J and Beaulieu P. Neuropharmacology. 2006 Jun;50(7):814–23 [23]
13	Acetaminophen (i.p.)	SR141716A SR144528 (i.p.)	The phenylbenzoquinone writhing test (in mice)	Analgesic effects of acetaminophen were not blocked by SR141716A and SR144528.	Acetaminophen in this test produced analgesia via a non–CB1, non–CB2 pain pathway	Haller VL, et al. Eur J Pharmacol. 2006 Sep 28;546(1–3):60–8 [24]
14	Arahidonic acid (i.v.)	Anandamide SR141716A (i.v.)	Tetrad model in mice (tests for analgesia, sedation, hypothermia and catalepsy)	Arachidonic acid produced the same profile of effects in tertrad as anandamide but neither substance was blocked by SR141716A.	The failure of SR141716A to antagonize the in vivo effects of anandamide suggested that non CB1R might be involved.	Wiley JL, et al. Life Sci. 2006 Dec 3;80(1):24–35 [25]
15	Ibu am–5 (the 6–methyl–pyridin–2–yl analogue of ibuprofen)		Tissue homogenate experiments or intact cell assays (in rats)	The compound Ibu am–5 inhibited rat brain anandamide hydrolysis by FAAH in a non–competitive manner. Ibu am–5 inhibited the binding of [3H]–CP55,940 to rat brain CB1Rs and to human CB2Rs more potently than ibuprofen.	The compound may be useful for the study of the therapeutic potential of combined fatty acid amide hydrolase–cyclooxygenase inhibitors.	Holt S, et al. Eur J Pharmacol. 2007 Jun 22;565(1–3):26–36 [26]
16	NS–398, indomethacin, acetaminophen, SC–560 (intracisternal)	WIN55,212–2 (intracisternal)	Intra–articular injection of formalin in temporomandibular joint (in rats)	An ineffective dose of WIN 55,212–2 in producing analgesia by intracisternal administration became effective following intracisternal administration of NS–398, indomethacin, acetaminophen, but not following SC–560.	Potentiation of WIN55212–2 with a selective COX–2 inhibitor, indomethacin, or acetaminophen was observed.	Ahn DK, et al. Pain. 2007 Nov;132(1–2):23–32. [27]
17	Acetaminophen (I pl)	AM251 AM630 (I pl)	Evaluation of mechanical allodynia and hyperalgesia in neuropathic rats	Acetaminophen decreased mechanical allodynia and hyperalgesia dose–dependently. These effects were inhibited by the administration of AM251 and AM630	The study suggested the implication of the endocannabinoid system in analgesia produced by acetaminophen	Dani M, et al. Eur J Pharmacol. 2007 Nov 14;573(1–3):214–5 [28]
18	Indomethacin (chronic treatment) (s.c.)	SWIN55,212–2 AM1241 Met–F–AEA (chronic treatment) (i.p.)	Streptozotocin (STZ)–induced neuropathic pain model	Chronic pretreatment with indomethacin progressively increased the analgesic effects of low doses of WIN 55,212–2, AM1241 and Met–F–AEA.	Indomethacin might potentiate the low doses of CB1 and CB2 agonists	Bujalska M. Pharmacology. 2008;82(3):193–200 [29]
19	Acetaminophen (p.o)	AM251 (i.p.) URB597 (i.p.) PMSF (s.c.)	thermal, mechanical and chemical pain tests	AM251 abolished the analgesic action of acetaminophen; inhibition of FAAH suppressed the analgesic effect of acetaminophen.	Two steps in acetaminophen–induced analgesia could be: FAAH–dependent metabolism of acetaminophen into AM404 and indirect involvement of AM 404 on CB1R stimulation.	Mallet C, et al. Pain. 2008 Sep 30;139(1):190–200. [30]
20	Diclofenac (s.c.)	URB597 (s.c.)	The acetic acid–induced writhing test (in mice)	Combinations of URB597 and diclofenac showed synergistic analgesic interactions.	According to isobolographic analysis, URB 597 and diclofenac acted synergistically in the writhing test	Naidu PS, et al. J Pharmacol Exp Ther. 2009 Apr;329(1):48–56. [31]
21	Acetaminophen (i.p.) morphine (s.c.), gabapentin(s.c.) and their combination	AM251 AM630 (s.c.)	The measure of hind paw hypersensitivity after acute compression of the mid–thoracic spinal cord	Pre–treatment with AM251 significantly diminished the analgesic effect of the acetaminophen + gabapentin combination. Both AM251 and AM630 reduced the efficacy of the acetaminophen + morphine combination.	Modulation of the endocannabinoid system might mediate the synergistic analgesic effects of acetaminophen combinations	Hama AT and Sagen J. Neuropharmacology. 2010 Mar–Apr;58(4–5):758–66. [32]
22	Aspirin (i.p.)	HU210 (i.p.)	hot–plate and formalin tests (in rats)	Low doses of HU210 significantly increased the analgesic effect of the sub–active dose of aspirin. SR141716A was ineffective per se and failed to modify analgesia induced by the HU210 plus aspirin combination.	Mutual potentiation of the analgesic effects of HU210 and aspirin might depend on an indirect participation of cannabinoid mechanism.	Ruggieri V, et al. Life Sci. 2010 Mar 27;86(13–14):510–7 [33]
23	R–flurbiprofen		Spinal cord microdialysis, after sciatic nerve injury in rats	R–flurbiprofen reduced glutamate release in the dorsal horn of the spinal cord evoked by sciatic nerve injury; also inhibited FAAH activity.	R–flurbiprofen improved the endogenous mechanisms to fend off the chronic neuropathic pain.	Bishay P, et al. PLoS One. 2010 May 13;5(5):e10628. [34]
24	Nimesulide (i.th.)	AM251 (i.th.)	Evoked responses of rat dorsal horn neurons in rats Spinal micro–dialysis	Spinal, but not peripheral, injection of nimesulide significantly reduced mechanically evoked responses of dorsal horn neurons that were blocked by AM251. Spinal levels of endocannabinoids were not elevated.	Responses to nimesulide were dependent on CB1R, without an implication of anandamide or 2–AG.	Staniaszek LE, et al. Br J Pharmacol.2010 Jun;160(3):669–76. [35]
25	Ibuprofen (i.p.) associated with acetaminophen (p.o)	AM281 (i.p.)	Acetic acid writhing test and hot plate test (in mice)	Additive analgesic effect in writhing test and potentiation in hot plate test. Adding AM281 the additive effect in writhing test is decreased and the potentiation in hot plate test disappeared	Influencing the cannabinoid system might be responsible for a part of analgesic effect of acetaminophen–ibuprofen combinations	Costescu M, et al Basic and Clinical Pharmacology and Toxicology. 2010, 107, Suppl. 1, 1: 243 [35]

## Discussions

We tried to systematize the results presented in the previous table by sorting the anti–inflammatory substances and their interactions with the cannabinoid system.

Indomethacin might interfere with the endocannabinoid system, as reported in some studies made by Burstein SH, et al. 1988 [12], Guhring H, et al. 2001[14], Anikwue R, et al. 2002 [15] and Bujalska M. 2008 [29]. Oral administration of indomethacin decreased the hiperalgesia produced by delta9–THC–a cannabinoid agonist, but in intrathecal administration did not influence the analgesic effects of HU 210–another cannabinoid agonist. In chronic oral administration delta9–THC decreased the effects of indomethacin, possibly by a pharmacokinetic mechanism (delta9–THC interfered the metabolism of indomethacin). The interference of indomethacin on the cannabinoid system is relatively controversial. Anikwue R, et al. 2002 [15] concluded that indomethacin might not react on the cannabinoid system, while Guhring H, et al. 2001[14] showed that indomethacin acted by means of the CB receptors. In his study, Bujalska M. 2008 [29] showed that indomethacin might potentiate the low doses of CB1 and CB2 agonists in a neuropathic pain model. Taking into account these studies, we can conclude that indomethacin interfere the cannabinoid system either by the CB receptors or by a pharmacokinetic mechanism.

Fowler CJ, et al. 1997 [13], Seidel K, et al. 2003 [18] and Guindon J, et al. 2006 [22] in their studies with ibuprofen, ibu am5 and flurbiprofen showed that all these substances inhibited FAAH. Ibuprofen acted synergistically with anandamide. This effect of ibuprofen was highlighted in experimental models for acute pain and also for neuropathic pain. Guindon J, et al. 2006 [22] concluded that ibuprofen potentiated the exogenous cannabinoids. Flurbiprofen, an ibuprofen derivative, intrathechally administrated proved an analgesic effect mediated by the endocannabinoid system, as result from Ates M, et al. 2003 [17], Seidel K, et al. 2003 [18] and Bishay P, et al. 2010 [34]. 

Some nonselective COX inhibitors, such as sulindac, ketoprofen and naproxen had been tested by Anikwue R, et al. 2002 [15], who showed that these substances did not act directly or indirectly on CB1 or CB2 receptors. On the other hand, aspirin proved to potentiate the effect of HU–210, a CB1 and CB2 receptor agonist (Ruggieri V, et al. 2010, [33]). After Naidu PS, et al. 2009 [31] diclofenac acted synergistically with URB 597 (a potent inhibitor of FAAH). 

Ketorolac, a selective inhibitor of COX1, had additive effects in association with WIN 55212–2, a nonselective cannabinoid agonist (Ulugol A, et al. 2006 [20]). However, other authors, like Anikwue R, et al. 2002 [15], proved that ketorolac did not act directly or indirectly on cannabinoid receptors

The selective COX2 agonists: NS–398, respectively rofecoxib, potentiated the action of cannabinoid agonists in acute pain models (Ahn DK, et al. 2007 [27]) or in neurophatic pain models (Guindon J and Beaulieu P. 2006 [23]). Celecoxib might not have a cannabinoid effect in the Anikwue R, et al. 2002 [15] study, while nimesulide showed an effect on CB1 receptors (Staniaszek LE, et al. 2010 [35]) without implication on anandamide or 2–AG levels. 

Out of all the substances included in the NSAIDs group, acetaminophen was studied the most regarding its interferences with the cannabinoid system mainly due to contradictory results. Hogestatt ED, et al. 2005 [19] showed that acetaminophen could be transformed in AM 404 in the central nervous system by FAAH. This metabolite is an agonist on TRPV1 receptors, a COX1 and COX2 inhibitor and inhibits the reuptake of anandamide, with an analgesic effect. There are some studies using acute pain models realized on animals  performed by Ottani A, et al. 2006 [21] and Mallet C, et al. 2008 [30] and other studies conducted on neuropathic pain models performed by Dani M, et al. 2007 [28] and Hama AT and Sagen J. 2010 [32] which sustain the existence of cannabinoid effects for acetaminophen. Other studies (Anikwue R, et al. 2002 [15], Haller VL, et al. 2006 [24]) had opposite results. Hama AT and Sagen 2010 [21] and Costescu M, et al 2010 [35] studied the association between acetaminophen and gabapentin, morphine or ibuprofen. They concluded that CB receptor blockers could antagonize the analgesic effects of these associations.  

## Conclusions

1. A clear antagonist, additive or synergic effect of NSAIDs–cannabinoid associations was not yet demonstrated. One of the causes for the variety of experimental results presented might be due to pharmacokinetic mechanisms, depending on the route of administration and the dose.
2. All the NSAIDs that inhibit COX2 can influence the cannabinoid system because a possible important degradative pathway for anandamide and 2–arachidonoyl glycerol might involve COX 2
3. Some NSAIDs have additional influences on the cannabinoid system either by inhibiting FAAH (i.e. ibuprofen, indomethacin, flurbiprofen, ibu–am5), or by inhibiting a possible intracellular transporter of endocannabinoids (i.e. acetaminophen).
